# The Liking for Hospitals

**Published:** 1920-10-09

**Authors:** 


### The Liking for Hospitals.

According to Dr. George .F Buchan, the Willesden Medical Officer of Health, the demand for hospital accommodation has been so great that it became necessary for the Council in March to restrict admission to the municipal hospital. The need for additional hospital accommodation in the district is urgent, as people generally prefer hospitals for the treatment of minor as well as more serious conditions. It is gratifying to note that of the 325 children under five years of age who were admitted last year to the municipal hospital fifty-one were so admitted at the request of private medical practitioners. It is difficult to estimate the number of beds which will be required in a community in the near future with the development of public opinion in the direction of hospital treatment, but it is probably no exaggeration to say that the number of hospital beds to be provided for all conditions other than mental diseases approximates 1 per cent. of the population, which would mean something like 1,700 for Willesden. There are in Willesden at present some 200 beds at the municipal hospital, 430 at the infirmary, 72 at the cottage hospital, 24 at St. Monica's Home, and 97 in use by tuberculous cases from Willesden, provided by the Middlesex County Council, or, approximately, 823 beds in all for Willesden people, showing a deficiency of about 877 beds. The proposal, therefore, before the Council to extend the municipal hospital by 200 beds is but a modest measure of the present needs of Willesden for additional accommodation.

